# Site-Specifically-Labeled Antibodies for Super-Resolution Microscopy Reveal *In Situ* Linkage Errors

**DOI:** 10.1021/acsnano.1c03677

**Published:** 2021-06-29

**Authors:** Susanna M. Früh, Ulf Matti, Philipp R. Spycher, Marina Rubini, Sebastian Lickert, Thomas Schlichthaerle, Ralf Jungmann, Viola Vogel, Jonas Ries, Ingmar Schoen

**Affiliations:** † Hahn-Schickard, Georges-Koehler-Allee 103, 79110 Freiburg, Germany; ‡ Laboratory for MEMS Applications, IMTEK, Department of Microsystems Engineering, University of Freiburg, 79110 Freiburg, Germany; § Cell Biology and Biophysics Unit, European Molecular Biology Laboratory (EMBL), 69117 Heidelberg, Germany; ^ Center for Radiopharmaceutical Sciences, Paul Scherrer Institute, 5232 Villigen, Switzerland; ∥ School of Chemistry, University College Dublin, Belfield, Dublin 4, Ireland; ∇ Department of Health Sciences and Technology, ETH Zurich, 8093 Zurich, Switzerland; ○ Faculty of Physics and Center for Nanoscience, Ludwig Maximilian University, 80539 Munich, Germany; # Max Planck Institute of Biochemistry, 82152 Martinsried, Germany; □ School of Pharmacy and Biomolecular Sciences, Irish Centre for Vascular Biology, Royal College of Surgeons in Ireland (RCSI), Dublin 2, Ireland

**Keywords:** antibodies, immunoglobulin G, transglutaminase, click chemistry, fluorescent probes, super-resolution microscopy, Monte Carlo simulations

## Abstract

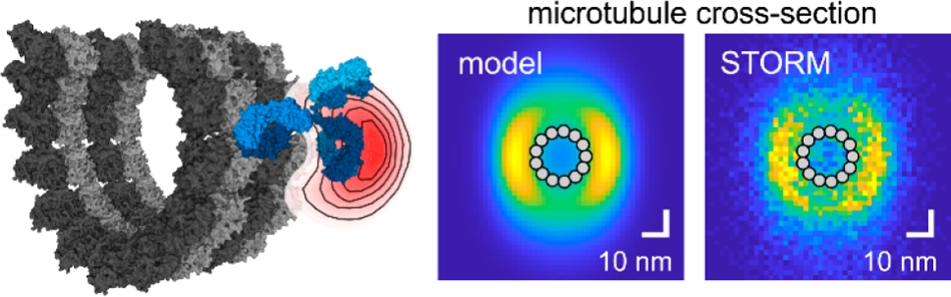

The precise spatial localization of proteins *in situ* by super-resolution microscopy (SRM) demands their targeted labeling. Positioning reporter molecules as close as possible to the target remains a challenge in primary cells or tissues from patients that cannot be easily genetically modified. Indirect immunolabeling introduces relatively large linkage errors, whereas site-specific and stoichiometric labeling of primary antibodies relies on elaborate chemistries. In this study, we developed a simple two-step protocol to site-specifically attach reporters such as fluorophores or DNA handles to several immunoglobulin G (IgG) antibodies from different animal species and benchmarked the performance of these conjugates for 3D STORM (stochastic optical reconstruction microscopy) and DNA-PAINT (point accumulation in nanoscale topography). Glutamine labeling was restricted to two sites per IgG and saturable by exploiting microbial transglutaminase after removal of N-linked glycans. Precision measurements of 3D microtubule labeling shell dimensions in cell lines and human platelets showed that linkage errors from primary and secondary antibodies did not add up. Monte Carlo simulations of a geometric microtubule-IgG model were in quantitative agreement with STORM results. The simulations revealed that the flexible hinge between Fab and Fc segments effectively randomized the direction of the secondary antibody, while the restricted binding orientation of the primary antibody’s Fab fragment accounted for most of the systematic offset between the reporter and α-tubulin. DNA-PAINT surprisingly yielded larger linkage errors than STORM, indicating unphysiological conformations of DNA-labeled IgGs. In summary, our cost-effective protocol for generating well-characterized primary IgG conjugates offers an easy route to precise SRM measurements in arbitrary fixed samples.

Super-resolution microscopy (SRM) enables the investigation of cellular structures with molecular resolution in the nanometer range.^1^ An ideal labeling strategy for SRM would achieve site-specific labeling with reporters as close as possible to the target such that the linkage error between epitope and reporter does not worsen the localization accuracy, while allowing for quantitative imaging approaches by a defined stoichiometry.^2^ These conditions are best met by genetic fusions of the target protein with a tag, like photoswitchable fluorescent proteins for PALM, or enzymes (SNAP, Halo) that mediate the conjugation with organic fluorophores or DNA handles for DNA-PAINT, respectively. While ectopic expression or CRISP-R Cas mediated genetic modification of many cell lines is straightforward nowadays,^3^ altered expression levels as well as the presence of the tag itself can lead to artifacts in the intracellular localization or to changed interactions between the target protein and its cellular environment.^4^ Moreover, some cells cannot be genetically modified *in vitro*, *e*.*g*., blood platelets, and tissue or liquid biopsies from patients for biomedical applications require alternative labeling approaches, namely, the usage of affinity tags.

Indirect immunofluorescence is the most common method to label target proteins in cell and tissue samples from patients. Highly target-specific primary antibodies are detected with secondary antibodies modified by reporter fluorophores (for STORM or STED) or DNA strands (for DNA-PAINT).^5^ Since nanometer localization precision can be achieved in single-molecule localization microscopy (SMLM), the secondary antibody contributes an additional linkage error between reporter and epitope, which has been estimated to be 10–15 nm,^5−7^ ultimately limiting measurement accuracy. Conceptually, linkage errors of antibodies are not well understood; while the antibodies usually bind their targets in a defined orientation resulting in a systematic offset between label and target (inaccuracy), the intramolecular flexibility and the random attachment sites of labels and/or secondary antibodies result in a spread of label positions (imprecision). These two distinct effects are often not distinguished in the literature, and the latter effect is difficult to disentangle from the localization imprecision (as given by the Crámer Rao lower bound, CRLB) and from uncompensated drift. Moreover, the quantification of the number of target proteins is complicated by the random number of modifications on the antibody.^5,20^ Although more and more fluorescently labeled primary antibodies are becoming commercially available, the catalogue is far from complete and lacks primary antibodies labeled with DNA handles for DNA-PAINT. Also, current commercial secondaries for DNA-PAINT are restricted to target species mouse and rabbit and do not offer customizable DNA sequences. Although several promising affinity reagents such as nanobodies, aptamers, or affimers have been developed in order to address these challenges,^6,8−10^ they rely on elaborate production techniques that are not routine in every laboratory environment, and their availability is still limited to a small number of targets. We propose that site-specific and stoichiometric labeling of immunoglobulins G (IgGs) with a simple protocol, which can be carried out in any lab, could offer a useful alternative for defined target labeling for SRM.

Antibody functionalization at specific sites and with a defined number of reagents is widely used for the synthesis of therapeutic antibody–drug conjugates (ADCs).^11^ These well-defined ADCs offer targeted delivery of drugs at better defined dosages and therefore create widened therapeutic windows in cancer chemotherapies.^12^ Established protocols include glycan trimming,^13,14^ localized lysine modification in the vicinity of histidine clusters,^15^ or labeling of specific glutamine residues that become accessible upon deglycosylation.^16−18^ These strategies hold promise to yield a homogeneous labeling of IgGs, have a high repeatability and minimal interference with the complementarity-determining region (CDR), and allow for the direct labeling of primary antibodies. These features could benefit SRM by achieving labeling closer to the epitope location, aiding colocalization analyses, denser sampling of cellular structures, and improved counting of proteins in supramolecular complexes. However, none of these approaches have been utilized for SRM to date, and their viability to modify nonhuman IgGs remains largely unexplored.

## Results/Discussion

### Site-Specific Antibody–Reporter Conjugation Strategy

**Scheme 1:**
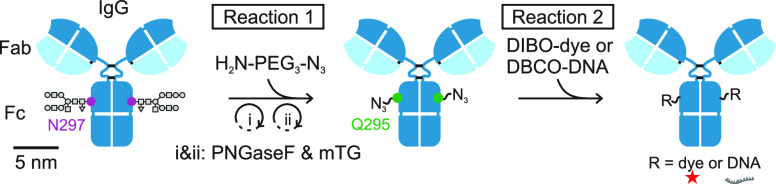
Site-specific labeling of IgG antibodies. In a first reaction, N-linked glycans are removed by PNGaseF, and only the available glutamines (Q) at position −2 relative to the deglycosylated sites are modified with H_2_N-PEG_3_-N_3_ catalyzed by microbial transglutaminase (mTG). In a second reaction, fluorophores(-DIBO) or ssDNA(-DBCO) are attached to the azide-modified antibodies using strain-promoted azide–alkyne cycloaddition (SPAAC).

In this work, we site-specifically label secondary and primary antibodies after deglycosylation using glutamine modification with azides (Scheme 1) following the pioneering work by Schibli and colleagues on ADCs.^17,18^ Such modified antibodies serve as a universal platform to couple different reporters *via* strain-promoted azide–alkyne cycloaddition (SPAAC).^19^ The functionalization protocol is based entirely on commercially available reagents. IgG antibodies are deglycosylated with the help of PNGaseF. Once the N-linked glycans are removed, microbial transglutaminase (mTG) catalyzes the cross-linking of amine-polyethylene glycol-azide (H_2_N-PEG_3_-N_3_) to the amide of the now-accessible glutamine at position −2 relative to the deglycosylated IgG site. In a final step, dibenzocyclooctyne (DBCO)-single-stranded DNA (ssDNA) or DIBO-Alexa Fluor (AF)647 is conjugated using SPAAC (Scheme 1, Supplementary Figure S1). All steps occur at neutral pH and under physiological salt concentrations. We combined the first two steps in a one-pot reaction to simplify the protocol. The intermediate and final products are purified by mild centrifugal filtration.

The conjugates were evaluated regarding the modification of a single glutamine per heavy chain with the help of mass spectrometry (Supplementary Figure S2) and regarding the degree of labeling (DOL) using UV–vis spectroscopy, which yields the average number of reporters per antibody. The conjugation of secondary antibodies raised in donkey resulted in two modifications per antibody for both DNA labels and fluorescent dye labels (Supplementary Table S1). The DOL moreover did not increase further with a higher excess of H_2_N-PEG_3_-N_3_ (80–300×) or of the reactive SPAAC probe (10–20×) (Supplementary Table S2). This result confirms previous reports using human IgG1 that found that mTG is specific to only one glutamine in each IgG heavy chain^17^ and that a sufficient excess of reagents can saturate these sites.

### Modification of Different IgG Subtypes from Different Species

**Figure 1:**
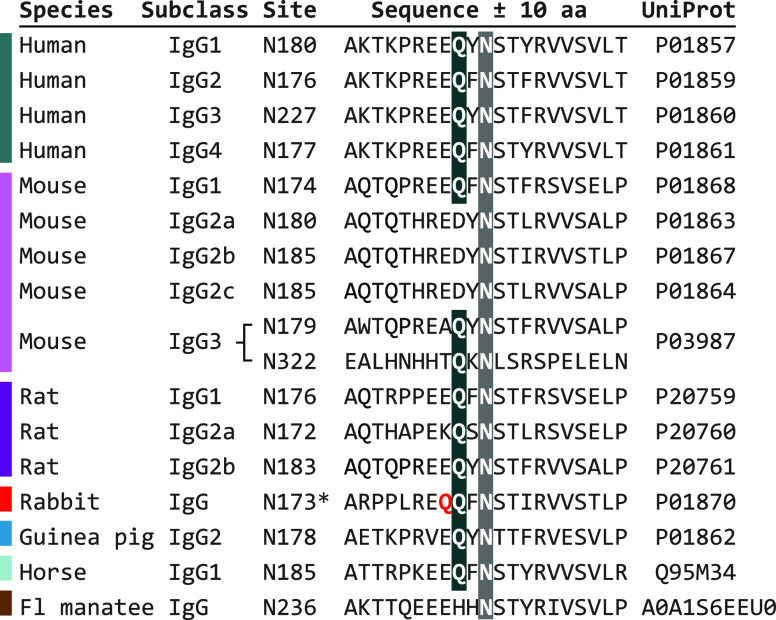
Sequence alignment and evaluation of potential modification sites for different IgG subclasses from different host species. Sequences were sourced from the UniProt database and aligned with respect to the N-linked glycosylation sites (gray background). The glutamine at position −2 (black background) is preserved across the majority of IgG subtypes and species for which sequence information was available. *Potential conflict with additional glutamine marked in red; Fl = Florida.

To explore the suitability of this method to modify IgGs from different host species in defined ways, we performed a sequence alignment (Figure 1) and tested our protocol for selected IgGs (Supplementary Table S1). The main N-linked glycosylation site is strictly preserved across species and IgG subtypes. The glutamine at position −2 with respect to this glycosylation site is preserved for all human IgG subclasses (IgG1, IgG2, IgG3, IgG4), mouse subclasses IgG1 and IgG3, all rat IgG subclasses (IgG1, IgG2a, IgG2b), in guinea pig (IgG2), and in equine species (IgG1 of horse or donkey). Accordingly, we found good compatibility with all tested donkey secondaries, two mouse IgG1 primaries, two rat IgG2a primaries, a mix of guinea pig IgG, and two humanized IgG1 primaries. The sequence analysis is also in agreement with previous successful modifications of human IgG2, IgG3, and IgG4 (P.R.S., personal communication). Mouse IgG3 has a second glycosylation site (N322) with an additional glutamine at position −2, which theoretically yields 4 instead of 2 possible modification sites per molecule (not tested). Rabbit IgG contains an additional glutamine at position −3 next to the conserved glutamine, and a sequence conflict was reported for the glycosylation site itself. Conjugation attempts with a rabbit primary or with rabbit secondaries showed negligible conjugation efficiency, which suggests that the glutamines are not accessible and/or modified. Manatee IgG and mouse IgG2 subtypes lack the buried glutamine and thus are not susceptible for labeling by this strategy, which was verified using one mouse IgG2b primary (Supplementary Table S1). No sequence information was found for goat or sheep IgGs; our conjugation attempts with goat secondaries resulted in ∼1 modification per IgG, which suggests either incomplete deglycosylation and/or a mixed population of different IgG subtypes. For compatible IgGs, no systematic differences were observed for DIBO–AF647 *vs* DBCO–DNA coupling efficiency. In summary, the conjugation method strictly depends on the conservation of the glutamine near the N-linked glycosylation site rather than on an IgG isotype or host species in general, and it is compatible with a wide range of antibodies.

### Microtubule Labeling Shell Dimensions in STORM

**Figure 2:**
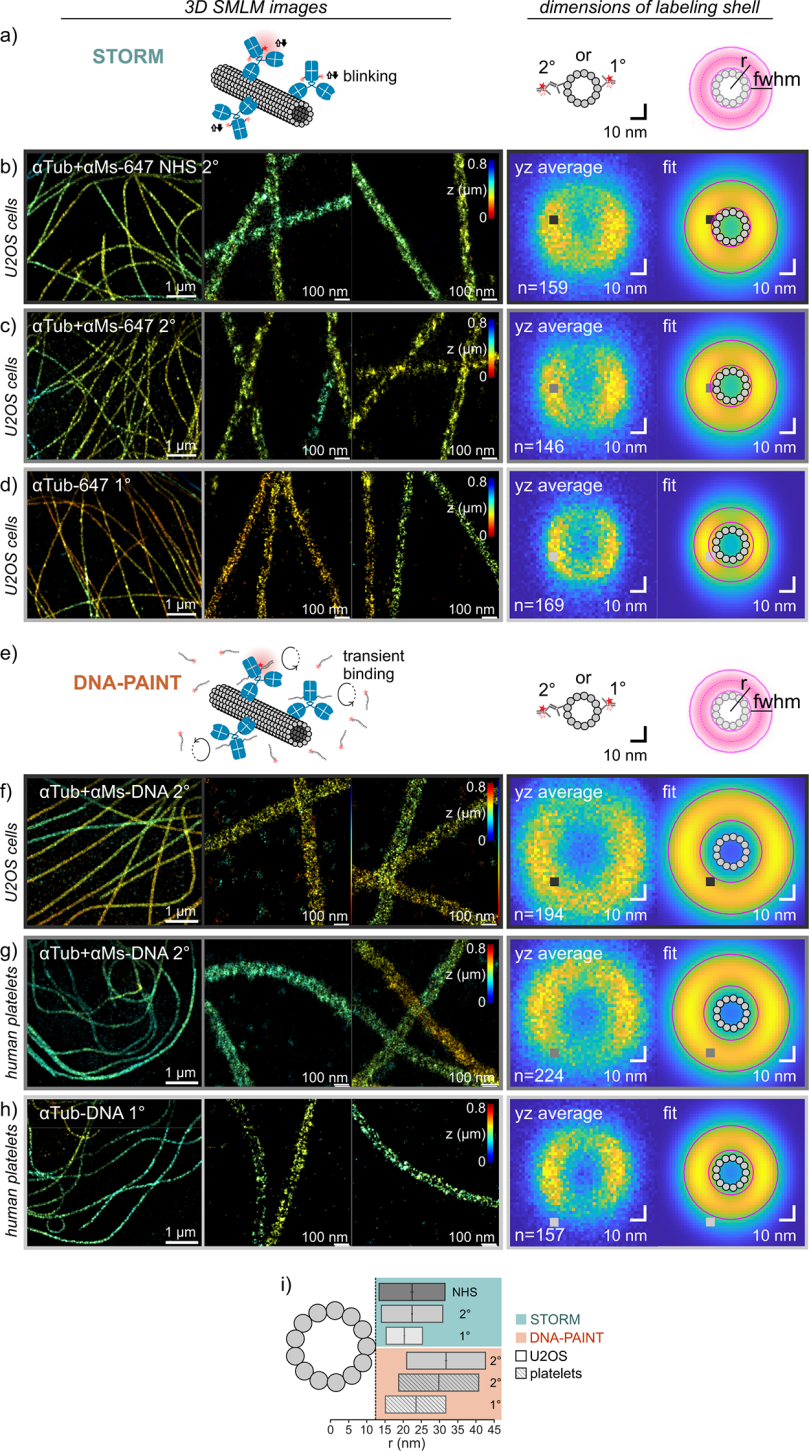
3D SMLM imaging of microtubule networks and determination of labeling shell dimensions for indirect and direct immunolabeling. (a–d) 3D STORM using Alexa Fluor 647 labeled antibodies in fixed U2OS cells. (a) Schematic (not to scale). (b) Indirect immunolabeling using a randomly labeled secondary donkey-anti-mouse (NHS 2°). (c) Indirect immunolabeling using a site-specifically-labeled secondary donkey-anti-mouse (2°). (d) Direct immunostaining using a site-specifically-labeled primary mouse-anti-α-tubulin (1°). (e–h) 3D DNA-PAINT using DNA-labeled antibodies in U2OS cells (f) and human platelets (g, h). (e) Schematic (not to scale). (f, g) Indirect immunolabeling using a site-specifically-labeled donkey-anti-mouse secondary (2°). (h) Direct labeling using a site-specifically-labeled primary mouse-anti-α-tubulin (1°). For all conditions, representative 3D SMLM images are shown (left). Labeling shell dimensions around microtubules were determined from averaged experimental *yz* cross-sections and fitted label distributions (right). The fitted label distribution was a Gaussian ring kernel (*r*: radius; *w*: full width at half-maximum) convolved with the localization precisions in *y* and *z*, respectively. (i) Comparison of center positions and widths of labeling shell dimensions for the different labeling strategies in b–d and f–h.

We benchmarked the fluorescent AF647-labeled antibodies for 3D STORM in fixed U2OS cells (Figure 2a–d) using microtubules as an *in situ* standard with known dimensions. We compared three cases: indirect immunolabeling with randomly labeled secondary antibodies (Figure 2b; NHS), indirect immunolabeling with our site-specifically-labeled secondary antibodies (Figure 2c; 2°), or direct immunolabeling with our site-specifically-labeled primaries (Figure 2d; 1°). The number of localizations per micron along microtubules decreased from the randomly labeled secondary (2159 ± 388 μm^–1^, mean ± std) over the site-specifically-labeled secondary (1634 ± 359 μm^–1^) to the primary (547 ± 136 μm^–1^), as expected from the effective number of fluorophores per bound primary. Directly labeled microtubules appeared distinctly thinner than indirectly labeled ones, and single antibodies were clearly discernible by the crisp appearance of localization clusters.

To quantify the linkage error that antibodies add to the microtubule dimension, we fitted the apparent dimensions of the antibody labeling shell around microtubules taking into account the thickness of the shell and in addition the blur by the localization precision (see Methods/Experimental). The averaged cross-sectional profiles and results are shown on the right of Figure 2b–d, and the derived dimensions are summarized in Table 1. The average outer radius of microtubules is 12.5 nm. The radius/center of the fluorophore-containing shell around microtubules was 22.5 ± 0.2 nm (best fit ±95% confidence interval) for randomly labeled secondaries, 22.5 ± 0.2 nm for site-specifically-labeled secondaries, and 20.2 ± 0.2 nm for site-specifically-labeled primaries. The thickness of the shell resulted as 18.1 ± 0.3, 16.9 ± 0.3, and 10.0 ± 0.5 nm, respectively. Dimensions were significantly different between the site-specifically-labeled 2° antibody and the 1° antibody (two-sided *F-*test; radius: *F* = 30.8, *p* < 0.0001; thickness: *F* = 27.7, *p* < 0.0001). Commercial secondary antibodies yielded the same radius (*F* = 0.2, *p* = 0.66) but a slightly thicker shell (*F* = 9.2, *p* = 0.0029) compared to the site-specifically-labeled secondaries, in agreement with previous findings, which found that immunostained microtubules were less well resolved when using antibodies with a higher degree of labeling.^20^ While these derived shell dimensions might be affected by nonstraight microtubules, imperfect drift correction, and/or imperfect registration, these errors should be comparable for different experiments. The comparison thus shows that direct immunofluorescence significantly decreases the offset to the epitope (thus, inaccuracy) by 2.3 nm and the spread of label positions (thus, precision) by ∼40% as compared to indirect immunofluorescence. Please note that the DOL of 1° and 2° antibodies was comparable due to our consistent labeling technique, which rules out the DOL as a potential confounding factor.^20^ The maximum linkage error (Table 1) thus was ∼19 nm for indirect and ∼13 nm for direct labeling. Importantly, the linkage errors of primary and secondary antibodies were not strictly additive. The obtained diameters of labeling shells (∼45 nm for secondary antibodies, ∼40 nm for primary antibodies) may be compared to previously reported apparent microtubules dimensions from 2D STORM images measured by the full-width-at-half-maximum (fwhm) of a single Gaussian fit to intensity line profiles perpendicular to the microtubule for secondary antibodies (59.5 nm,^20^ 61.7 nm^8^), primary antibodies (54.0 nm^8^), or secondary nanobodies (37.5 nm,^21^ 39.3 nm^8^) or by measuring the peak-to-peak distance of a double Gaussian fit using secondary antibodies (35 nm^22^) or secondary nanobodies (∼32 nm,^23^ 30–40 nm^24^). Please note that these derived values still contain systematic errors due to the *z*-projection, localization imprecision, and oversimplified fitting models.

**Table 1:** Measured Dimensions of the Microtubule Labeling Shell of Different Secondary (NHS, 2°) or Primary (1°) Antibody–Reporter Conjugates Using STORM (“647”) or DNA-PAINT (“DNA”), Respectively^a^

cells	antibody labeling	shell radius *r* (nm)^b^	thickness *w* (nm)^b^	minimum linkage error^c^ (nm)	maximum linkage error^d^ (nm)
U2OS	αTub + αMs-647 NHS 2°	22.5 ± 0.18	18.1 ± 0.27	1.0 ± 0.32	19.1 ± 0.32
U2OS	αTub + αMs-647 2°	22.5 ± 0.22	16.9 ± 0.34	1.6 ± 0.41	18.5 ± 0.41
U2OS	αTub-647 1°	20.2 ± 0.24	10.0 ± 0.48	2.7 ± 0.54	12.7 ± 0.54
U2OS	αTub + αMs-DNA 2°	31.8 ± 0.14	21.6 ± 0.19	8.5 ± 0.24	30.1 ± 0.24
human platelets	αTub + αMs-DNA 2°	29.2 ± 0.16	21.4 ± 0.21	6.0 ± 0.26	27.4 ± 0.26
human platelets	αTub-DNA 1°	23.5 ± 0.20	16.7 ± 0.28	2.7 ± 0.34	19.4 ± 0.34

a Parameters were obtained from fits shown in Figure 2.

b Best fit parameters ±95% confidence intervals.

c The minimum linkage error was calculated using the formula (*r* – 12.5 nm) – 0.5*w*.

d The maximum linkage error was calculated using the formula (*r* – 12.5 nm) + 0.5*w*.

### Microtubule Labeling Shell Dimensions in DNA-PAINT

Next, we used the ssDNA-labeled antibodies for DNA-PAINT imaging of microtubules *in situ* in U2OS cells or in spread human platelets (Figure 2e–h). Similar to STORM, directly labeled microtubules (1°) appeared distinctly thinner compared to indirect immunolabeling (2°), both in the images themselves and in terms of the averaged cross-sections. The radius of the microtubule labeling shell resulted as 31.8 ± 0.14 nm for indirect DNA-PAINT in U2OS cells (reanalysis of previously published data^25^) and 29.2 ± 0.16 nm in platelets (*F* = 80.9, *p* < 0.0001) and similar thicknesses of 21.6 ± 0.19 and 21.4 ± 0.21 nm, respectively (*F* = 3.5, *p* = 0.0628) (Table 1). Dimensions obtained by direct DNA-PAINT were 23.5 ± 0.20 nm for the radius and 16.7 ± 0.28 nm for the thickness and thus highly significantly smaller than those obtained by indirect DNA-PAINT (radius: *F* = 129.0, *p* < 0.0001; thickness: *F* = 57.7, *p* < 0.0001). The microtubule diameters of indirect DNA-PAINT (∼59–63 nm) may be compared with previous 2D DNA-PAINT measurements of the peak-to-peak distance of a double Gaussian fit to the perpendicular intensity line profile (∼47 nm^26^). Please note that in a *z*-projection the peak intensities move inward and a fit tends to underestimate the true labeling shell diameter. The maximum linkage error in our DNA-PAINT measurements thus was ∼27–30 nm for indirect and ∼19 nm for direct labeling, again emphasizing that the linkage errors of primaries and secondaries are not strictly additive.

Unexpectedly, the label shell dimensions in DNA-PAINT were significantly larger than those in STORM, in terms of both radius and thickness of the labeling shells, both for indirect labeling (radius: *F* = 137.83, *p* < 0.0001; thickness: *F* = 44.4, *p* < 0.0001) and for direct labeling (radius: *F* = 47.7, *p* < 0.0001; thickness: *F* = 30.2, *p* < 0.0001) (Figure 2i). This was consistently observed in different cell types and on different microscopes, for fitting of pooled cross-sections (Figure 2) as well as of individual microtubule segments (Supplementary Figure S3). Of note, AF647 fluorophores on secondary antibodies reached close to the microtubule surface, whereas DNA docking strands on secondary antibodies were depleted from around the microtubule (see Figure 2i and minimum linkage errors in Table 1). While an underestimation of the localization precision, which was smaller in DNA-PAINT as compared to STORM, might lead to an overestimation of the fitted shell thickness, it has little impact on the fitted radius (Supplementary Figure S4). The same strict filtering criteria regarding deviations from the experimental point spread function were applied, which rules out systematic experimental localization errors. The design of the DNA-PAINT imager strand positioned the fluorophore at the antibody-proximal end of the docking strand, only a C5 linker length (between DBCO and ssDNA, *ca*. 0.5 nm) away from the epitope, similar to DIBO-AF647. We thus conclude that technical aspects of the two SMLM imaging approaches cannot fully explain the observed difference in the label distribution around microtubules in STORM *versus* DNA-PAINT experiments.

### Linkage Errors in Monte Carlo Simulations of Immunolabeled Microtubules

**Figure 3:**
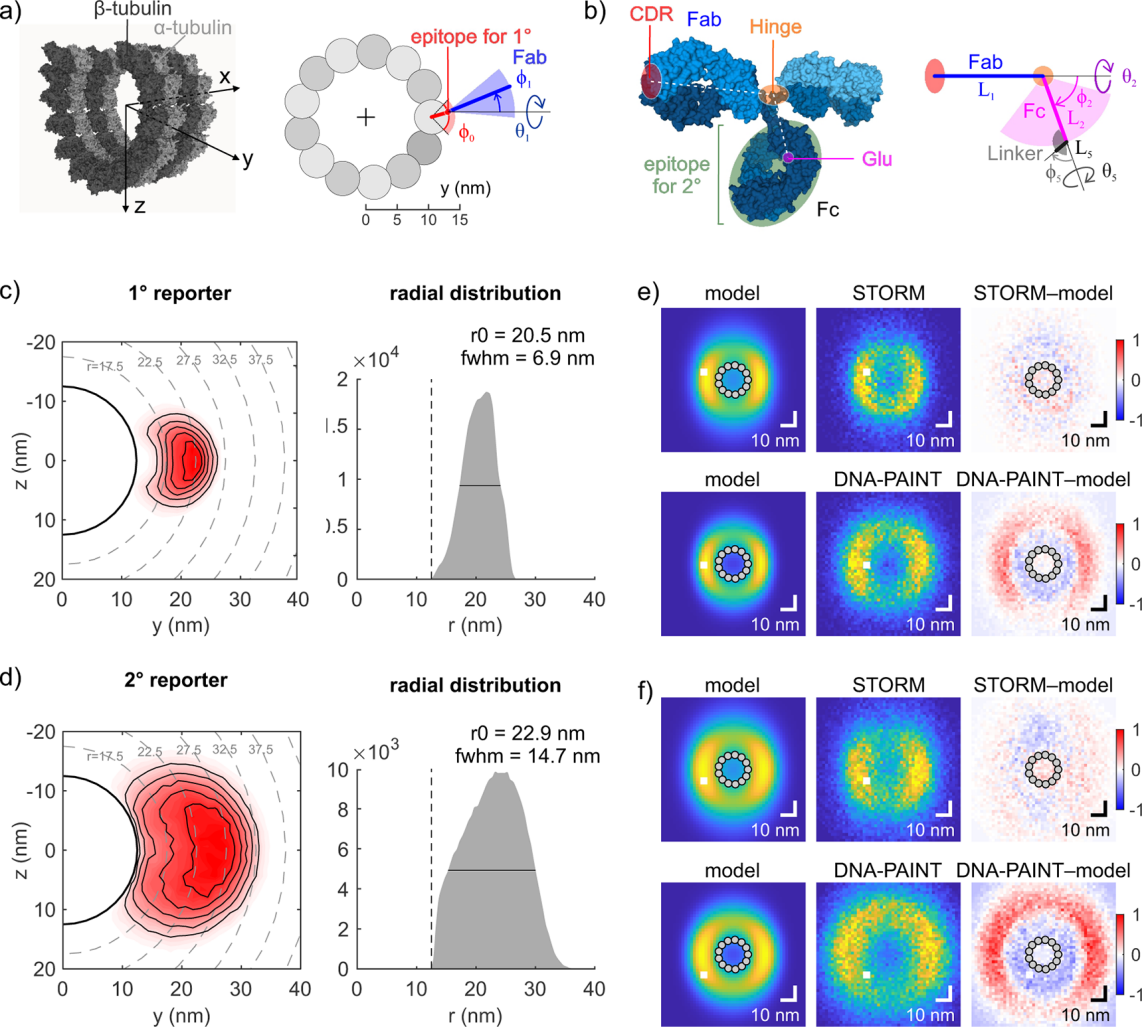
Monte Carlo simulations of antibody conformations at microtubules. (a) Location of the epitope of the anti-α-tubulin antibody in our geometric model (right) based on the cryo-EM structure of a microtubule (left; PDB 5SYF, ref (27); molecular surface rendered using Mol*, ref (33)). (b) Each IgG molecule was modeled by two segments corresponding to Fab and Fc fragments connected by a flexible hinge region. Right: Parametrization of Fab, Fc, and linker segments of the labeled primary antibody. Left: Dimensions were based on the crystallographic structure of IgG2a (PDB 1IGT, ref (30), visualization by Mol*). While the location of the modification is precisely known (Glu), the binding sites of polyclonal secondary antibodies are assumed to be evenly distributed over the Fc region. For a schematic of indirect immunolabeling, see Supplementary Figure S5c). Simulated reporter distribution for primary antibodies. Left: *yz* cross-section. Right: Radial distribution. (d) Simulated reporter distribution for primary plus secondary antibody complexes. Panels as in (c). (e) Comparison of reporter distributions for primary antibodies between simulations (left; convolved with the localization and experimental imprecision) and experiments (middle: pooled cross-sections as in Figure 2). Right: Normalized residuals of the difference between experiment and model. Top: STORM. Bottom: DNA-PAINT. (f) Comparison of reporter distributions for secondary antibodies between simulations and experiments. Panels as in (d).

To better understand the spatial distribution of reporters around immunolabeled microtubules, we performed Monte Carlo simulations of immunolabeled microtubules using a geometric model that takes advantage of the known attachment site of the reporter at IgGs due to our site-specific labeling strategy (Figure 3, Supplementary Tables S3 and S4). The primary antibody binds an epitope on α-tubulin at the outer surface of a microtubule, which was modeled based on cryo-EM data^27^ (Figure 3a). Fab and Fc antibody segments were simulated as rigid bodies joined by a flexible hinge, which is a validated and widely accepted assumption.^28,29^ Segment lengths were determined from a mouse IgG2a crystal structure,^30^ while hinge flexibility was modeled according to the measured conformational distribution of IgG molecules in solution^31^ (Figure 3b). The binding sites of polyclonal secondary antibodies were assumed to be evenly distributed over the Fc region of the primary antibody. The reporter was attached to Glu295 *via* a flexible C5 linker. Steric clashes were excluded by restricting angles between neighboring segments, obeying a minimum distance between non-neighboring segments, and treating the microtubule as impenetrable (see Methods).

The label distribution of simulated primary antibodies peaked at 20.5 nm distance from the microtubule center and had a fwhm of 6.9 nm (Figure 3c). The simulated label distribution for indirect immunolabeling peaked at 22.9 nm and showed a much larger spread of 14.7 nm (Figure 3d). While the peak positions of the label distributions vary with the choice of the unknown epitope location on the microtubule (Supplementary Figure S6) and the unknown rigidity of the Fab-epitope orientation (Supplementary Figure S7) by up to ±1.4 nm, the by *ca*. 2.4 nm larger peak distance and the by 7.8 nm substantially wider distribution for indirect compared to direct immunolabeling were robust against these parameter changes. This can be rationalized by the following mechanism: The flexible hinge region within the primary antibody largely randomizes the direction into which its Fc segment and accordingly also the secondary antibody protrude. As a consequence, the secondary antibody mainly adds to the spread and much less to the distance of the labels from the target. This explanation for nonadditive errors of primary and secondary antibody layers based on molecular conformational flexibility shares some similarities with a proposed explanation based on mere error propagation,^32^ which, however, does not account for the orientation bias of the primary antibody nor distinguish between inaccuracy (systematic offset) and imprecision (spread).

### Benchmarking of SMLM Results against Simulations

The relative differences between simulated primary and secondary label distributions in terms of peak position and width favorably compare to the relative differences between the label distributions that resulted from STORM data (Table 1). Moreover, simulated and experimental (STORM) peak positions agreed quantitatively, at least within the uncertainty caused by the choices of unknown parameters (see above). The experimental fwhm was larger by ∼3 nm compared to the simulated width, which could arise from nonstraight microtubules or from an imperfect fit of the axis direction of individual microtubules. When convolved with the experimental localization uncertainties, the simulated cross-sections of primary and secondary label distributions matched the STORM cross-sections very well (Figure 3e,f). We conclude that the results from STORM experiments match the theoretical predictions.

Systematic differences were seen when comparing DNA-PAINT cross-sections to simulations, with a considerable outward shift of the measured labeling shell (Figure 3e,f). Technical reasons could be excluded since STORM imaging of indirectly labeled microtubules using DNA-labeled primary and AF647-labeled secondary antibodies yielded similar results (Supplementary Figure 8a) as DNA-PAINT of indirectly labeled microtubules (Figure 2f,g). We thus proposed that DNA-labeled (primary or secondary) antibodies assumed different conformations than the AF647-labeled antibodies or the simulated antibodies. A possibility is that negative charges of DNA led to a repulsive interaction and a radial alignment of antibodies. Simulations of more straight antibody conformations shifted the peak of the radial label distributions outward and depleted the labels from the microtubule vicinity as in the experiments (*cf*. Figure 2i); however, they substantially reduced the width of the label distribution and thus systematically underestimated the measured shell thicknesses, for both direct and indirect labeling (Supplementary Figure 8b). Since a mere reorientation could not fully explain the larger shells, we hypothesized that the contour length of the antibody was increased. Deglycosylation is known to render IgGs more prone to chemical or thermal denaturation.^34,35^ We speculate that removing positively charged glycans and adding negatively charged DNA in close proximity could potentially destabilize the Fc structure and cause partial unfolding, which would increase the contour length and flexibility. Far-UV circular dichroism (CD) spectra showed negligible changes upon IgG deglycosylation but a more negative ellipticity at 217 nm of DNA–IgG conjugates (Supplementary Figure 8c), comparable to signal changes resulting from other denaturation pathways.^36,37^ Since it was not possible to obtain further experimental evidence for IgG partial unfolding upon DNA conjugation, the underlying reason for the larger labeling shells in DNA-PAINT remained incompletely understood.

## Conclusions

Site-specific and quantitative functionalization of antibodies with fluorescent dyes or ssDNA with the help of mTG and subsequent click reactions was highly consistent for the tested IgGs, as opposed to common NHS labeling, which varies more strongly from batch to batch and with the number of reactive lysine residues on different antibodies. Our work thus significantly extends the scope of the original approach^18^ by demonstrating its applicability to a range of IgGs from different species and their applications to SRM. Similar functionalization results for a range of other compatible IgG subtypes and primary antibodies can be expected based on sequence analysis except mouse IgG2 and rabbit IgG (Figure 1 and Supplementary Table S1). As a note of caution, the protocol is incompatible with the presence of gelatin, BSA, or primary amines in storage buffers, as these interfere with the enzymatic coupling step, a minor problem that can be overcome by prepurification *via* protein A/G resin and/or dialysis.

Our precise determination of labeling shell dimensions based on averaged microtubule cross-sections and the modeling of antibody complex conformations allowed an unequivocal interpretation of linkage errors when using antibodies for SRM, summarized in Figures 2i and 3. The excellent agreement between results from STORM and Monte Carlo simulations validates the dimensions of, and the considerable flexibility within, the antibodies. Mapping of the exact epitope location of the used anti α-tubulin antibody could eliminate the remaining uncertainty and further improve theoretical predictions. Secondary antibodies increased the maximum linkage error consistently by 50% less compared to primary antibodies themselves, as a direct consequence of the higher flexibility of the 1°+2° antibody complex than of the 1° alone (Figure 3). Importantly, a purely additive model in which labeling shells behave like impenetrable monolayers is neither consistent with these data nor plausible because the labeling in reality is too sparse to induce interactions between neighboring antibodies. Lastly, the larger outer dimensions in DNA-PAINT compared to STORM require more straightened conformations of 1°+2° antibody complexes and even exceed the theoretical maximum epitope–reporter distance in 1° antibodies. We speculate this could be related to partial loss of IgG quaternary structure upon DNA conjugation.

Our results highlight an unconventional application area for ADC conjugation chemistries for SRM, by providing a straightforward two-step protocol. Our demonstration of labeling and imaging of microtubules in platelets exemplifies the usage of conjugates with cell lines that cannot be genetically engineered and is directly translatable to patient samples. The site-specific modification of glutamines in the Fc region avoids the modification of lysine residues at the CDR and thus provides an alternative approach in cases where amine labeling results in a reduction of binding affinity, a problem variably encountered when labeling primary antibodies. Since the protocol needs neither special reagents nor equipment and comes at *ca*. 20 € per reaction (Supplementary Table S5), it is easily established in most molecular biology laboratories, as no complicated and costly genetic engineering of antibodies is required. Its adoption could thus benefit many super-resolution microscopists.

## Methods/Experimental

### Antibody Labeling

Functionalization reactions were carried out with 50 μg of antibody. The following IgGs were used for SRM: donkey anti-rabbit IgG (Jackson Immuno Research, ref: 711-005-152); donkey anti-mouse IgG (Jackson Immuno Research, ref: 715-005-151); mouse anti-β-tubulin (ThermoFisher Scientific, 32-2500). Other IgGs used for testing the conjugation protocol are listed in Supplementary Table S1. First, 50 μg of antibody was incubated with 0.3 U PNGaseF (Roche, ref: 11365193001), 0.6 U mTG (Zedira, ref: T001), and an 80× molar excess of H_2_N-PEG_3_-N_3_ (click chemistry tools/Jena Bioscience, ref: CLK-AZ101-100) in a one-pot reaction at 37 °C overnight on a shaker at 300 rpm. The unreacted H_2_N-PEG_3_-N_3_ was removed using a centrifugal concentrator (GE Healthcare; Vivaspin 500, 50 kDa nominal molecular weight cutoff) by topping up with PBS to 500 μL and spinning at 7000 rcf for 5 min; this was repeated with freshly added PBS two more times before the sample was recovered. Please note that the dilution with PBS and the relatively large final filtrate volume of ∼40 μL due to reduced centrifugation speed/time ensure that the antibody concentration never significantly exceeded the original concentration (∼1 mg/mL) as a precautionary measure to prevent aggregation. The azide-modified antibodies were then incubated with a 10× molar excess of either DIBO-Alexa Fluor 647 (for STORM; Life Technologies, ref: C10408) or DBCO–DNA (for DNA-PAINT; sequence: DBCO-5′-TTATACATCTA-3′, Biomers) for 2.5 h at RT. The unreacted click reagent was removed using a centrifugal filter as above. Antibodies were stored in PBS pH 7.4 with 1% bovine serum albumin (BSA) and 0.05% sodium azide at 4 °C under exclusion of light for up to 3 months (secondaries) or without BSA at −20 °C for up to 3 years (primaries). Note that pH affects enzyme activity; if the IgG storage buffer is not at physiological pH (∼7.4), pH adjustment prior to the reaction is advisable since a test reaction carried out at pH 6.0 yielded a reduced DOL of ∼0.4–0.5. Should the antibody be prone to aggregation upon deglycosylation, the addition of up to 0.05% Tween 20 to stabilize the antibody in solution is tolerated by the enzymes and does not interfere with reactions.

### DOL Characterization

UV–vis absorption on a Nanodrop was used to calculate the average degree of labeling. The DOL for the AF647-conjugated antibodies was determined from the measured absorption values (Nanodrop) at 280 nm (A280) and 650 nm (A650) using the formula DOL = (A650/239 000) × 210 000/(A280 – 0.03 × A650), where 0.03 is the correction factor for AF647 absorption at 280 nm, 239 000 is the molecular extinction coefficient of AF647 at 650 nm, and 210 000 is the molecular extinction coefficient of IgG at 280 nm. The DOL for DNA-conjugated antibodies was determined from the measured absorption values at 280 nm (A280) and 260 nm (A260) using the formulas DOL = cAb/cDNA with cAb = (A280 – A260 × 0.61)/(210 000 – 0.55 × 0.61 × 210‘ 000) and cDNA = (A260 – cAb × 210 000 × 0.55)/142 000, where 0.61 is the correction factor for DNA absorption at 280 nm, 0.55 is the correction factor for protein absorption at 260 nm, 210 000 is the molecular extinction coefficient of IgG at 280 nm, and 142 000 is the molecular extinction coefficient of the P1 handle single-stranded oligo sequence at 260 nm.

### Sample Preparation for SMLM

U2OS cells (ATCC HTB-96) were cultured in phenol-red-free DMEM supplemented with 10% fetal calf serum (FCS), NEAA, and Glutamax. Cells were seeded on methanol/HCl-cleaned 24 mm round #1.5 coverslips 2 days before fixation. Washed platelets were purified from whole blood. Blood was obtained from healthy consenting volunteers in line with the declaration of Helsinki and national regulations following ethical approval (RCSI Research Ethics Committee 1394 and 1504). Washed platelets were spread on cleaned 20 mm round #1.5 fibrinogen-coated coverslips in Tyrode’s buffer containing 1.8 mM CaCl_2_ and 5 μM ADP for 1 h. Both U2OS cells and platelets were briefly washed, extracted in 0.3% glutaraldehyde and 0.25% (v/v) Triton X-100 in cytoskeletal buffer (CB: 10 mM MES pH 6.1, 150 mM NaCl, 5 mM EGTA, 5 mM glucose, 5 mM MgCl_2_) for 1–2 min, fixed with 2% glutaraldehyde in CB for 10 min, washed, and quenched with 0.1% (w/v) sodium borohydride in PBS for 7 min. Fixed samples were permeabilized (PBS containing 0.1% (v/v) Triton X-100, 0.5% (w/v) bovine serum albumin) for 15 min, washed, blocked (3% BSA in PBS) for 40 min, stained with primary antibodies (10 μg/mL in PBS with 3% BSA) at 4 °C overnight, washed, stained with secondary antibodies (15 μg/mL in PBS with 3% BSA) for 2 h, washed, postfixed (2% paraformaldehyde in PBS), washed, and stored (PBS containing 0.05% sodium azide) at 4 °C in the dark for up to 3 weeks.

### Quantification of Apparent Microtubule Dimensions

Individual regions of interest were defined along microtubules, and 200–300 nm long lines were manually drawn in SMAP.^38^ The microtubule axis was refined by registering the 3D localizations that lay within <60 nm of the drawn line by least-squares fitting of a hollow 3D cylinder to the data. The registered data were then projected along the cylinder axis. Projected localizations were binned into 2 × 2 nm bins and fitted by a Gaussian ring kernel with ring radius *r* and sigma σ that was convolved with the anisotropic localization precisions σ_*y*_ (which here stands for the mean of σ_*x*_ and σ_*y*_) and σ_*z*_ in the lateral and the *z*-directions, respectively, in the following way. First, to account for the non-normal distribution of CRLB values in either *y* or *z*, projected localizations were binned according to their CRLB values in a two-dimensional array, and a compound anisotropic filter kernel was constructed by adding up two-dimensional Gaussians using the binned σ_*y*,CRLB_ and σ_*z*,CRLB_ values as widths and the bin counts as relative weights. Second, to account for the residual uncompensated drift, a two-dimensional Gaussian using the independently determined uncertainties σ_*y*,Drift_ and σ_*z*,Drift_ (see Supplementary Methods) was used as a second filter kernel. The only free fitting parameters were thus *r* and σ. For the thickness of the labeling shell, we used the fwhm = 2.35 σ of the underlying label distribution. About 150–250 sections along straight parts of microtubules from 2 to 5 cells/separate acquisitions were evaluated per condition.

### Monte Carlo Simulations

Monte Carlo simulations were performed and analyzed in MATLAB 2020a (Mathworks). Length measurements of rigid Fab and Fc segments were obtained from the crystal structure of mouse IgG2a (PDB 1IGT)^30^ (see Supplementary Table S3). Possible epitope locations on α-tubulin were assessed from the cryo-EM structure of Taxol-stabilized microtubules (PDB 5SYF).^27^ Structures were taken from the RSCB database and measured in Mol*^33^ (see Supporting Information). Starting from the epitope location (0, *y*_0_, *z*_0_) and orientation ϕ_1_ on the microtubule, a kinked chain was constructed characterized by linear segments of lengths *L*_*i*_ and rotational angles ϕ_*i*_ and θ_*i*_ around the *x*-axis and *y*-axis, respectively, that uniquely defined their orientation with respect to the preceding segment (*i* > 1) or to the global coordinate system (*i* = 0; see Figure 3a). Direct immunolabeling was represented by 3 segments (CDR-Hinge, Hinge-Glu, Glu-Reporter; see Figure 3a,b), indirect immunolabeling by 5 segments (1°CDR-1°Hinge, 1°Hinge-2°epitope, 2°CDR-2°Hinge, 2°Hinge-2°Glu, 2°Glu-Reporter; see Supplementary Figure S5). Lengths were either randomly sampled from a finite set of lengths, as measured within the crystal structure, or uniformly picked from a continuous range within defined boundaries (Supplementary Table S4). DBCO–DNA and DIBO–AF647 linkers were modeled as having the same characteristics. A total of 300 000 (primary) or 400 000 (1°+2°) conformations were simulated. Steric hindrance by the microtubule was enforced by demanding that all chain kink positions lie outside of the microtubule (*y*_*i*_^2^ + *z*_*i*_^2^) > (*y*_0_^2^ + *z*_0_^2^). Steric hindrance between antibody segments was implemented by restricting kink angles ϕ_*i*_ of sequential segments and by enforcing that the distance between any pair of points lying on two nonsequential segments was larger than *d*_min_. Conformations that did not meet these criteria were neglected. The radial distance of the label from the microtubule center was determined from the *y* and *z* coordinates of the chain end point. Contour plots were created by 1.25 × 1.25 nm binning in *y* and *z* and subsequent 2d interpolation. For comparison with experimental cross-sections, the radial label density was revolved around the origin and convolved with the experimental (CRB plus residual drift) precisions in *y* and *z*. For determining the residuals, the convolved cross-section was rescaled (offset and magnitude) to best match the experimental cross-section (least-square fit), subtracted, and normalized by the maximum intensity of the rescaled cross-section.

### Statistical Analysis

To investigate the potential significance of fitted labeling shell parameters (radius and thickness), we employed a two-sided *F*-test for pairwise comparisons by setting the standard deviations to  times the 95% confidence intervals of fitted parameters, where *n* denotes the number of cross-sections that were pooled for the fit. *p-*values were calculated from *F-*values using the degrees of freedom for the numerator (=1) and denominator (=0.5(*n*_1_ + *n*_2_ – 2)). Comparable *p-*values were obtained by comparing the fit results of individual microtubule cross-sections (Supplementary Figure S3) for two conditions by an unpaired *t*-test.

## Supporting Information Available

Experimental methods on mass spectrometry, SMLM setups, SMLM imaging, the determination of residual experimental errors, and circular dichroism spectroscopy measurements; Supplementary Figures S1 to S8; Supplementary Tables S1 to S6 (PDF)
