# Supplementary material for: Site-Specifically-Labeled Antibodies for Super-Resolution Microscopy Reveal In Situ Linkage Errors
Source: ACS Nano. 2021 Jun 29;15(7):12161–70. doi: 10.1021/acsnano.1c03677 (PMC8320235; doi:10.1021/acsnano.1c03677)
Supplement: Supplementary file 1 — nn1c03677_si_001.pdf [file nn1c03677_si_001.pdf]

Supplementary Information

Site-Specifically Labeled Antibodies for Super-Resolution Microscopy Reveal *In Situ* Linkage Errors

Susanna M. Früh, Ulf Matti, Philipp R. Spycher, Marina Rubini, Sebastian Lickert, Thomas Schlichthaerle, Ralf Jungmann, Viola Vogel, Jonas Ries, Ingmar Schoen*

* corresponding author: ingmarschoen@rcsi.ie

Table of contents:

Experimental section .... page 2

Supplementary figures .... page 5

Supplementary tables .... page 13

Supplementary references .... page 17

Experimental Section

LC-ESI-MS analysis. Spectra were measured on a Waters LCT Premier mass spectrometer. 10 µg of antibody was incubated with DTT (20 mM final concentration) at 37 °C for 30 min. Samples were chromatographed on an Aeris WIDEPORE XB-C18 column (3.6 µm, 100 mm × 2.1 mm; Phenomenex) heated to 80 °C and eluted at a flow rate of 0.5 mL/min using the following gradient: 0−1 min, 10−20% A; 1−30 min, 20−60% A; 30−31 min, 60−10% A (solvent A: 1:1 acetonitrile:isopropanol, 0.1% formic acid; solvent B: water, 0.1% formic acid). The eluent was ionized using an electrospray source (ESI+). Data were acquired with MassLynx 4.1 and deconvolved using MaxEnt1.

Microscopy setups for STORM and DNA-PAINT. SMLM imaging was performed at room temperature (21 °C) on two home-built setups of similar layout but located in different labs. Setup 1 at EMBL, as previously described in Li *et al.*,[1] was used for STORM and DNA-PAINT in U2OS cells (Fig. 2 b,c,d,f) while Setup 2 at RCSI was used for DNA-PAINT in platelets (Fig. 2 g,h). Setup 1 contained a high numerical-aperture (NA) oil-immersion objective (160×/1.43-NA; Leica, Wetzlar, Germany), a laser combiner (LightHub; Omicron-Laserage Laserprodukte, Dudenhofen, Germany) with Luxx 405 and 638 lasers, and an EMCCD camera (Evolve512D; Photometrics, Tucson, AZ, USA). The effective pixel size was 140 nm. Setup 2 contained a high numerical-aperture (NA) oil-immersion objective (60×/1.49-NA; APON60XOTIRF; Olympus, Hamburg, Germany), iBeam smart 405 and 640 lasers (Toptica Photonics AG, Gräfelfing, Germany), and an sCMOS camera (Orca Flash v4; Hamamatsu Photonics Ltd, UK). The effective pixel size was 108 nm. In both setups, lasers are passed through a speckle reducer (LSR-3005-17S-VIS; Optotune, Dietikon, Switzerland) and coupled into a multimode fiber (M105L02S-A; Thorlabs, Newton, NJ, USA) whose output is magnified by an achromatic lens, filtered to remove fiber-generated fluorescence (390/482/563/640 HC

Quad; AHF, Tübingen, Germany), and imaged into the sample.[2] Laser and camera triggering were controlled by an FPGA (Mojo; Embedded Micro, Denver, CO, USA). A closed-loop focus lock system was implemented, using total internal reflection of a near-infrared laser (iBeam smart 785, Toptica Photonics AG) at the coverslip-buffer boundary, its detection by a quadrant photodiode, and the output voltage to control the objective piezo (P-726.1CD; Physik Instrumente Ltd, Bedford, UK). The fluorescence emission was filtered by a bandpass filter (700/100, AHF) and the PSF engineered using an astigmatic lens. Microscopes were controlled using µManager 2.0gamma and EMU.[3]

Super-resolution imaging. Labeled samples were mounted in a chamber containing STORM imaging buffer (0.2 M Tris pH 8.2, 10 mM NaCl, glucose oxidase, catalase, 4% (w/v) D(+) glucose, 35 mM mercaptoethanolamine). The power of the UV 405 nm activation laser was automatically adjusted to maintain a constant number of activations over the duration of the movie. Between 70'000-200'000 frames were acquired at 50 s^{-1}, depending on when the activation ceased. DNA-PAINT was performed in PAINT imaging buffer (1x PBS, 0.2 M Tris pH 8.2, 0.5 M NaCl) containing ca. 1 nM P1 imager strands. Movies were acquired for 200'000-600'000 frames at 5 s^{-1}. Single molecule switching movies were fitted using an experimental 3D point spread function.[1] Localizations were filtered by log-likelihood ratio >-1.5 and a lateral localization precision better than 15 nm (STORM) or 8 nm (DNA-PAINT) before visualization and further analysis. FRC measures of microtubule STORM images were taken from 0.3 x 1-2 µm long sections along isolated microtubules for each condition. All SMLM data were corrected for drift in x,y,z. All analysis was done using the software package SMAP[4].

Quantification of residual experimental uncertainties. Imperfect drift correction, as well as other remaining uncertainties (noise from hardware autofocus lock, deviations of the actual PSF from the fitted spline-interpolated PSF, *etc*) contribute to a lower effective localization precision as predicted by the CRLBs of the Maximum Likelihood Estimation (MLE) fit.

Tetraspec beads (100 nm, Invitrogen) were adsorbed onto a coverslip at a density of 15-30 beads per 25 x 25 µm field of view. To mimic the measurement duration and typical photon count of STORM (or DNA-PAINT) acquisitions, respectively, the exposure time was set to 20 ms (200 ms), the repetition rate to 5 s^{-1} (0.5 s^{-1}), the number of frames to 15’000 (40’000), and the laser power to ca 2.5% of that in SMLM experiments. Fitting and drift correction were performed on ungrouped data with the same settings as for SMLM movies. For each single bead, the standard deviations of localization positions around the median x, y, and z positions were determined, yielding the experimental uncertainties in xy and z. The ideal xy and z uncertainties for each bead were determined by taking the mean of the localizations’ Cramer Rao Lower Bound (CRLB). The residual imprecision was calculated from the median real and ideal uncertainties of all beads by $\sigma_{Drift} = (\sigma_{real}^2 - \sigma_{CRLB}^2)^{0.5}$ for xy and z separately. When using settings similar to STORM, the residual imprecision resulted as 2.9±0.5 nm and 4.3±0.5 nm for lateral and axial localization, respectively (mean ± standard deviation from 3 independent acquisitions). For settings similar to DNA-PAINT, the residual imprecisions resulted as 3.7±0.6 nm for x or y and 7.1±0.8 nm for z. The residual drift thus was the dominant imprecision in DNA-PAINT data.

Circular dichroism measurements. Donkey anti-mouse IgGs were DNA-conjugated (DOL 1.9; see above), treated with PNGase only, or left unmodified. Far-UV (200-250 nm) CD spectra of IgGs (0.11 mg/ml in PBS) were measured on a Jasco J-810 spectropolarimeter with 0.5 nm data pitch, 50 nm/min scan speed and an accumulation of 6. PBS was measured as a reference and subtracted from PNGase-treated and unmodified IgG spectra. DBCO-ssDNA (1.47 µM in PBS) was measured as a reference to account for CD signals from ssDNA and subtracted from the spectrum of the DNA-conjugated IgG.

Supplementary Figures

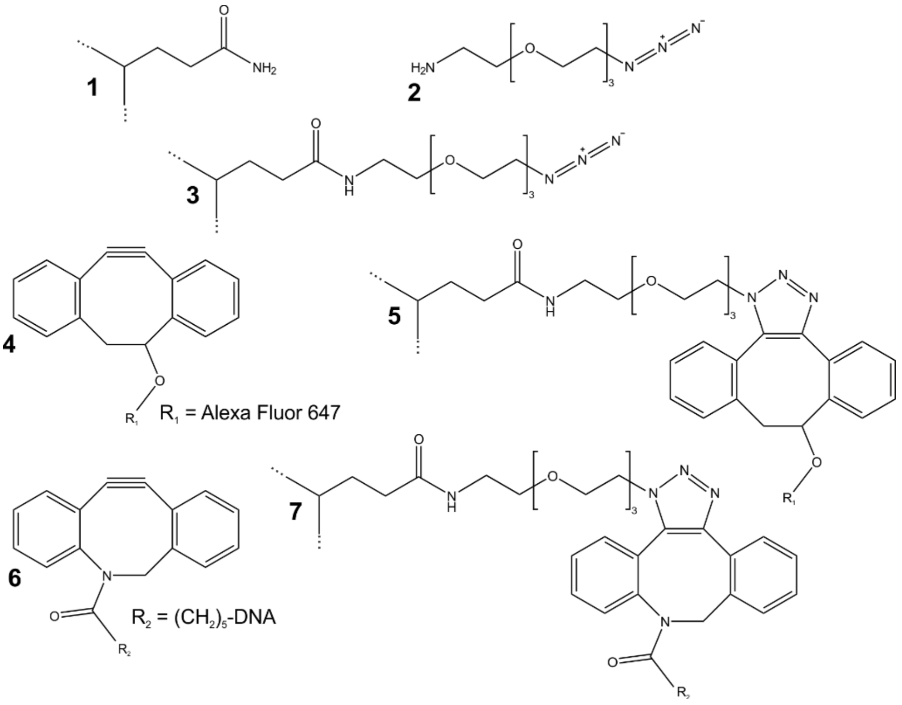

Figure S1. Reagents and reaction products. 1: glutamine residue; 2: amine-PEG$_3$-azide bifunctional linker; 3: product of mTG-mediated conjugation of 2 to 1; 4: DIBO-AF647; 5: product of SPAAC-mediated conjugation of 4 to 3; 6: DBCO-ssDNA; 7: product of SPAAC-mediated conjugation of 6 to 3.

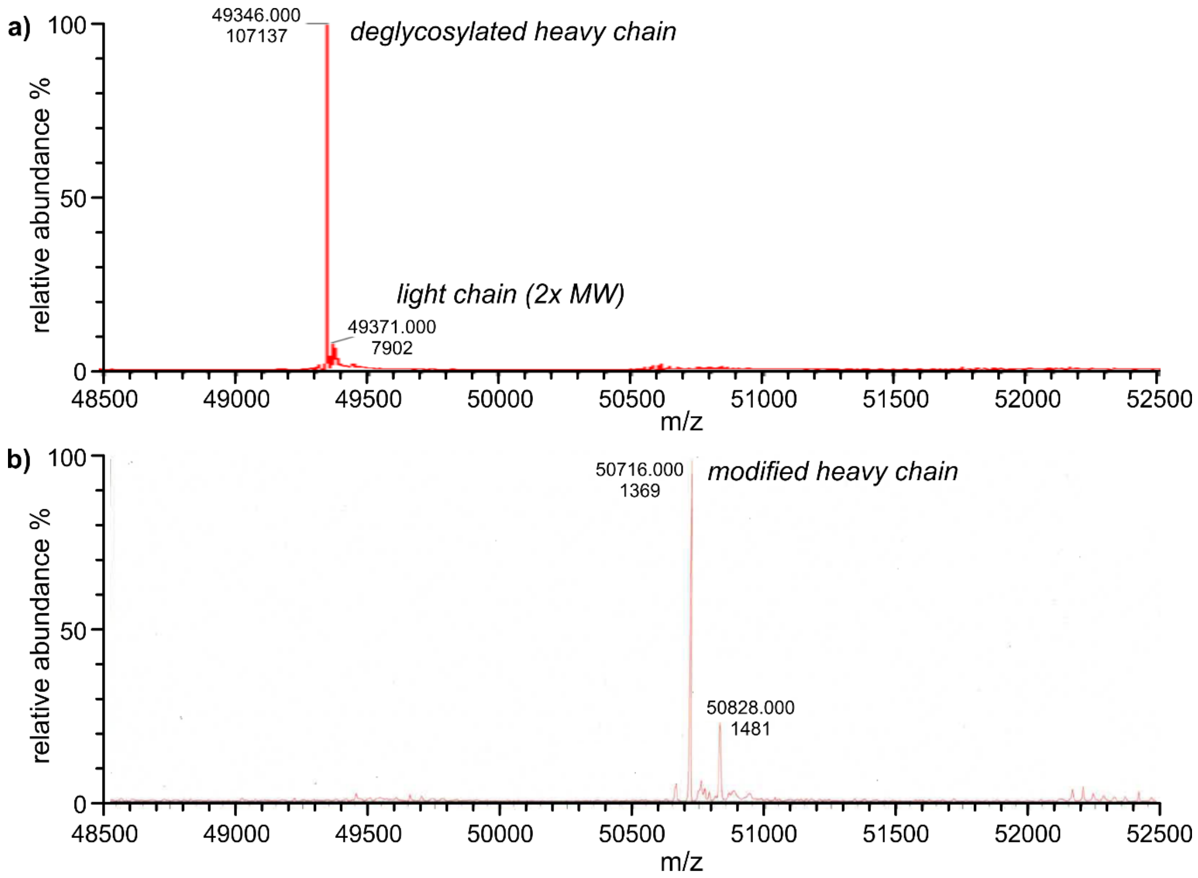

Figure S2. TOF-MS ES+ spectra of intermediate and end products of the IgG modification reaction. The monoclonal chCE7 antibody (see Table S1) is shown as an example after a) PNGaseF treatment and after b) modification with H_2N-PEG_3-N_3 and DIBO-Alexa Fluor 647, which contributes to a difference in mass between non-glycosylated heavy chain and modified heavy chain of ~1370 Da.

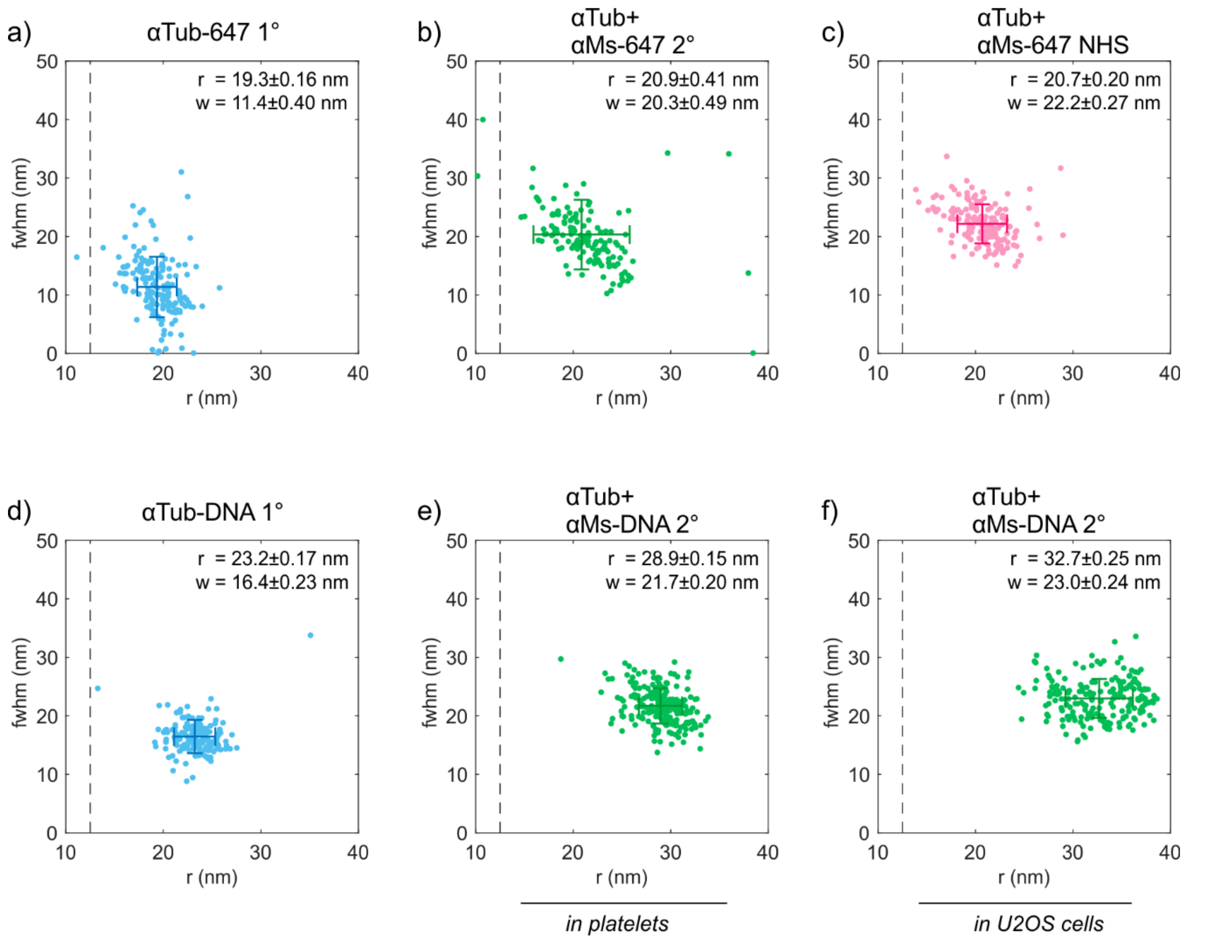

Figure S3. Quantification of microtubule dimensions from individual, 200-300 nm long sections imaged by a)-c) 3D STORM or d)-f) 3D DNA-PAINT in either a)-c), f) U2OS cells or d)-e) human platelets. Shown are the radius r and thickness (full width at half maximum, fwhm) of the labeling shell as derived from cross-sectional fits. Compared to fits of pooled cross-sections (cf. Figure 2 in the main manuscript), the fitting tended to yield increased shell thicknesses, but overall confirmed the differences between direct (a+d) or indirect (b,c,e,f) labeling, as well as between STORM and DNA-PAINT.

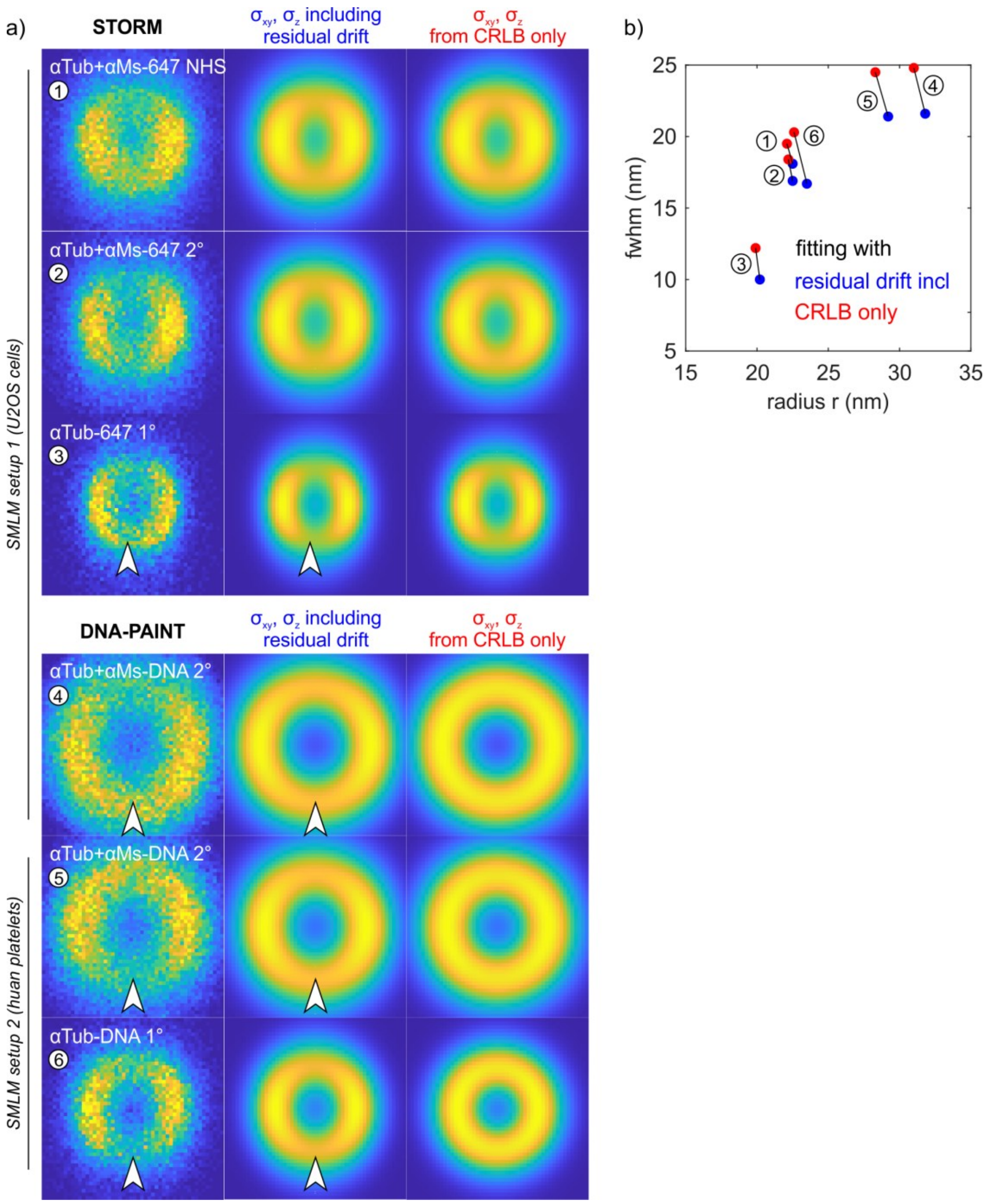

Figure S4. Dependence of the goodness of the fit of the microtubule labeling shell cross-sections on including the imprecision from residual uncompensated drift *vs* accounting for the CRLB only. a) Qualitative comparison of fitted labeling densities. The diminished signal at top and bottom of the MT in experimental data (left, arrowheads) is reproduced by larger σ_z as obtained by including the residual drift imprecision (middle) while the CRLB values alone lead to a more homogenous appearance (right), especially for DNA-PAINT. The CRLB is thus an underestimate, especially for DNA-PAINT where the CRLBs are ~half of those in STORM. b) Using the CRLB underestimate for fitting led to a slight underestimation of the fitted radius r (by 0.3-0.4 in STORM, 0.8-0.9 nm in DNA-PAINT) and a more substantial overestimation of the fitted width w (by 1.4-2.2 nm in STORM, 3.3-3.6 nm for DNA-PAINT).

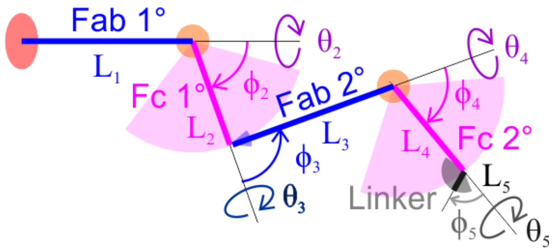

Figure S5. Definition of lengths and angles of the segments in a 1ary+2ndary antibody sandwich. For the choice of parameters, see Table 2 in the main text.

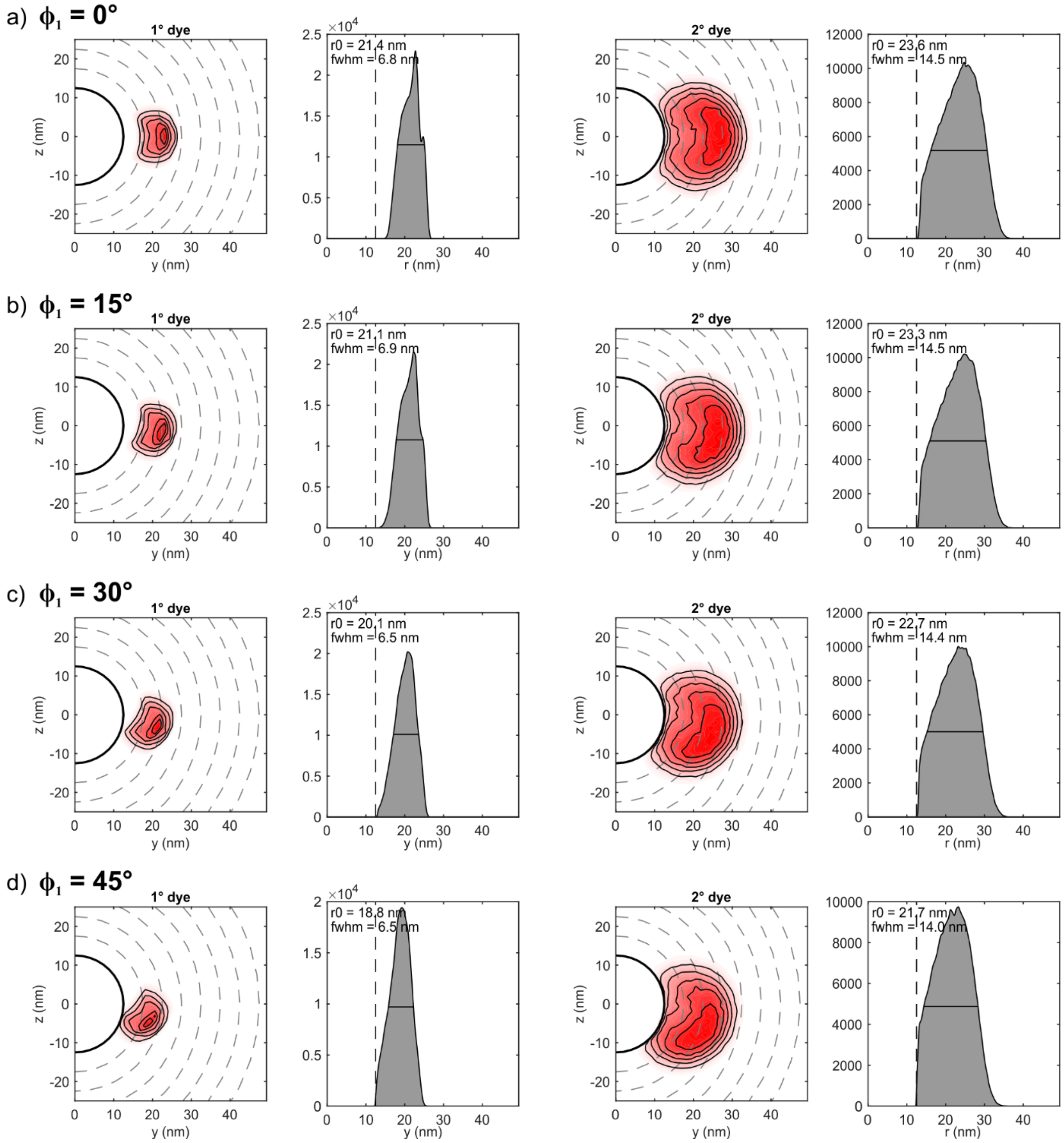

Figure S6. Simulated reporter distribution of primary (left) or secondary (right) antibodies around microtubules depending on the location and orientation of the epitope on α-tubulin. The location/orientation is parameterized by the single parameter ϕ_1 (see Figure 3a and Table 2 in the main text). *Left panels:* Contour plots of the reporter location density projected onto the plane of the microtubule cross-section. *Right panels:* radial distribution of the reporter. Variation of ϕ_1 from straight a) 0° to increasingly oblique positions b) 15°, c) 30°, and d) 45° in the groves of the microtubule. Accordingly, the label moved closer to the microtubule while the spread remained largely unchanged.

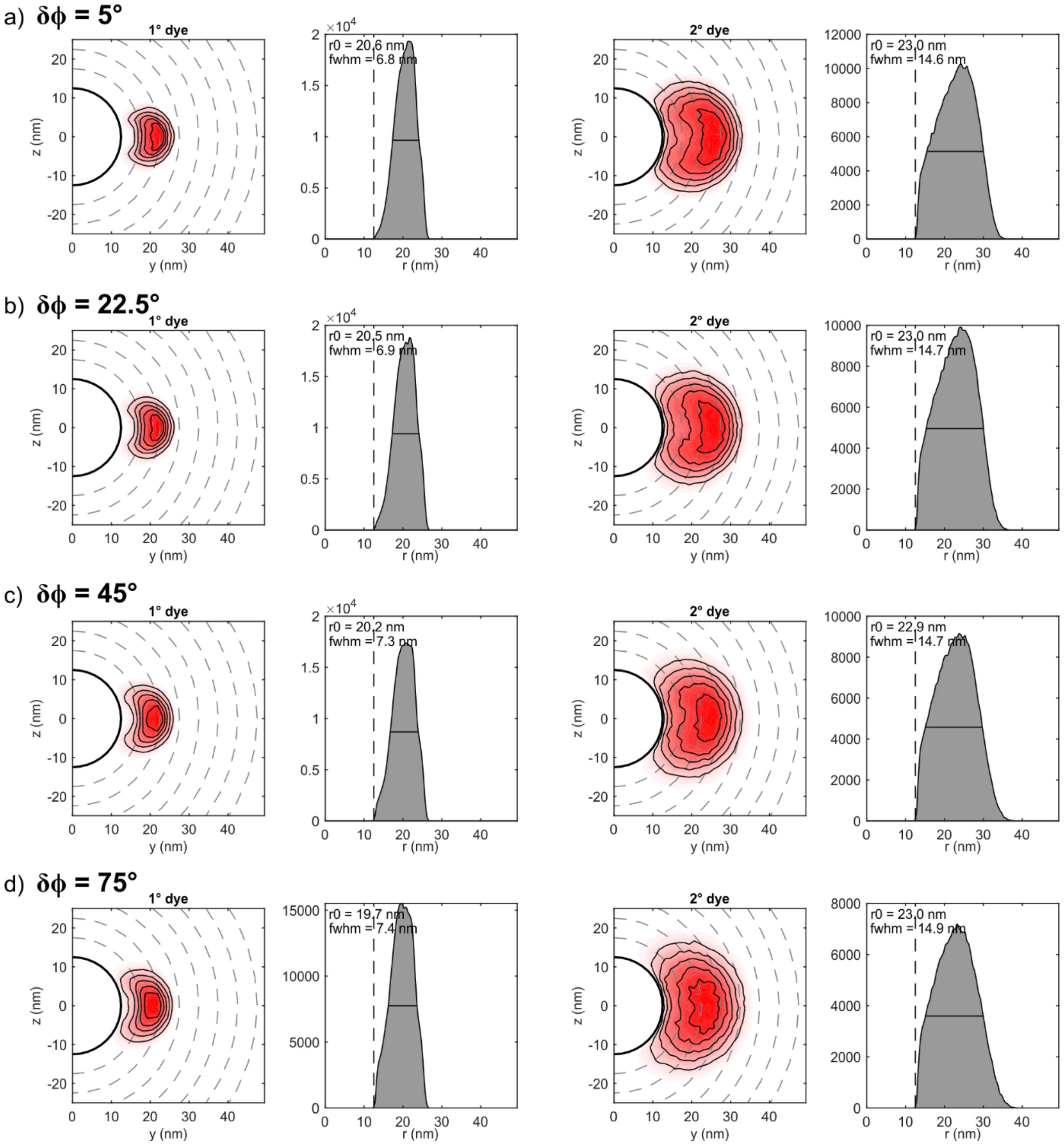

Figure S7. Simulated reporter distribution of primary (left) or secondary (right) antibodies around microtubules depending on the flexibility of the Fab-epitope binding orientation. The flexibility is parameterized by the parameter δϕ (see Figure 3a and Table 2 in the main text). Left panels: Contour plots of the reporter location density projected onto the plane of the microtubule cross-section. Right panels: radial distribution of the reporter. Variation of δϕ from rigid a) 5°, b) 22.5° to flexible c) 45°, d) 75°. With increasing flexibility of the Fab-epitope orientation, the primary antibody label moved closer to the microtubule; notably, no change was observed for peak of the secondary label distribution. In both cases, a minor widening of the radial distribution resulted from an increased Fab-epitope flexibility.

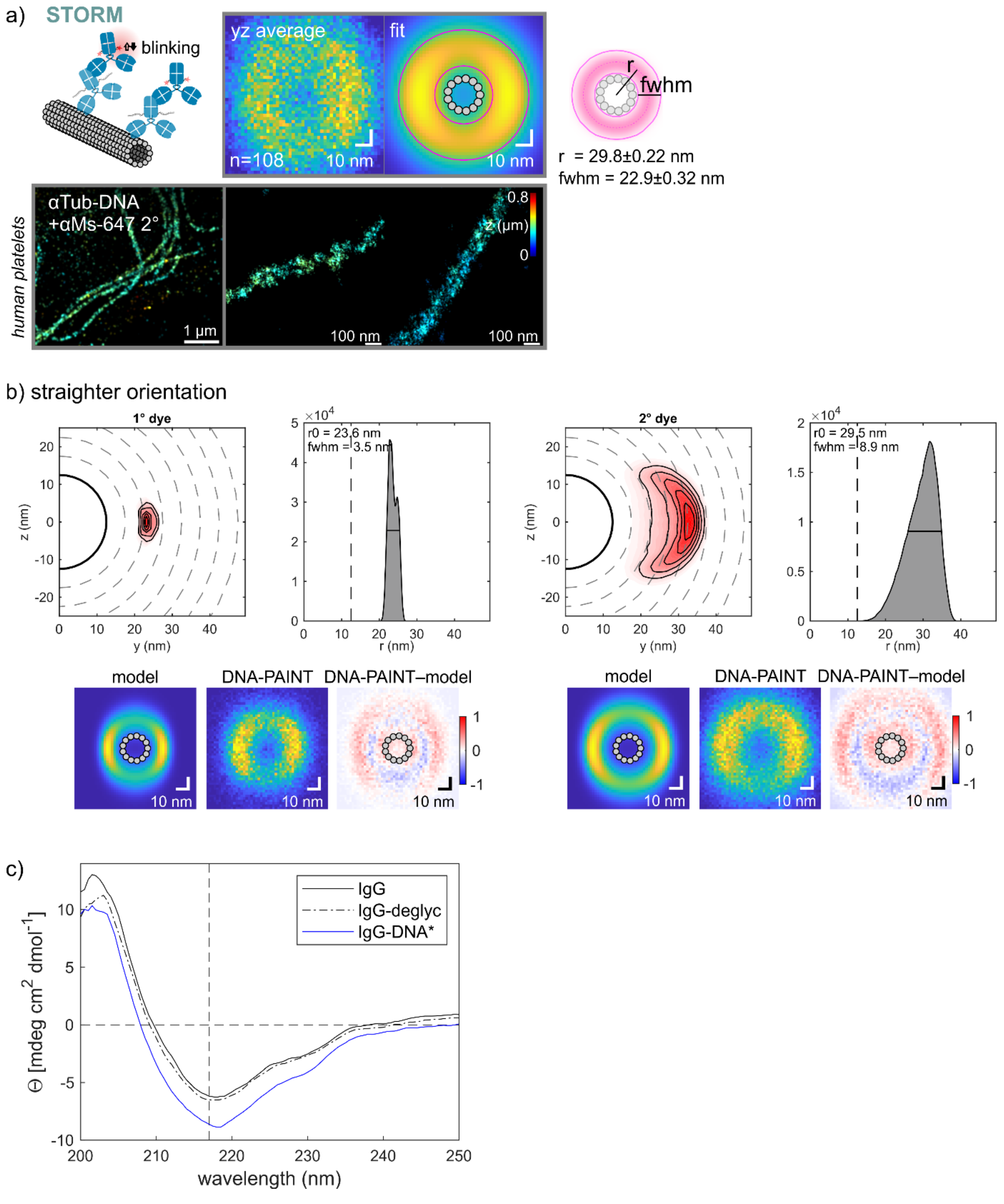

Figure S8. Investigation of potential reasons for larger and thicker labeling shells obtained by DNA-PAINT. a) STORM of indirectly labeled microtubules using DNA-labeled primary antibodies yielded substantially thicker and larger labeling shells than with unlabeled primaries. b) Adjusting model parameters for straighter antibody labeling complexes to match the peak position of the experimentally determined label distribution leads to a drastically decreased labeling shell thickness, as opposed to a thicker labeling shell seen in DNA-PAINT. c) Circular dichroism (CD) spectrogram of unconjugated donkey anti-mouse IgG, deglycosylated IgG, and DNA-conjugated IgG. The latter was corrected for the CD spectra of an equivalent amount of DBCO-DNA along. A more negative ellipticity was observed in the region around 217 nm for the DNA-IgG conjugate, in line with reported changes for chemical denaturation of IgGs.[5]

Supplementary Tables

Table S1. Tested secondary and primary antibodies. DOL of antibody-Alexa Fluor 647 or antibody-ssDNA(P1) conjugates for various IgG isotypes.

Host species	IgG isotype	clonal	target	Vendor / source	clone / order number	modification	DOL	# tested reactions	remark
Donkey	IgG1	poly	Mouse IgG H+L	Jackson	715-005-151	AF647	2.0±0.1	8	used in Fig. 2a
Donkey	IgG1	poly	Mouse IgG H+L	Jackson	715-005-151	P1	1.9±0.2	5	used in Fig. 3b
Donkey	IgG1	poly	Rabbit IgG H+L	Jackson	715-005-152	AF647	1.7±0.3	6	
Donkey	IgG1	poly	Rabbit IgG H+L	Jackson	715-005-152	P1	1.8±0.2	4	
Donkey	IgG1	poly	Goat IgG H+L	Jackson	711-005-147	AF488	1.4±0.1	3	
Mouse	IgG1	mono	beta Tubulin	ThermoFisher Scientific	32-2600	AF647	1.2	1	used in Fig. 2b
Mouse	IgG1	mono	beta Tubulin	ThermoFisher Scientific	32-2600	P1	1.8	1	used in Fig. 3c
Mouse	IgG1	mono	alpha Tubulin	ThermoFisher Scientific	MS-581-P1ABX	AF647	1.9	1	
Mouse	IgG2b	mono	Lamin A/C	Santa Cruz Biotechnology	sc-7292X	AF647	0.1	1	incompatible (cf. Fig. 1)
Rat	IgG2a	mono	alpha Tubulin	ThermoFisher Scientific	MA1-80017	AF647	2.2	1	
Rat	IgG2a	mono	alpha Tubulin	ThermoFisher Scientific	MA1-80017	P1	1.9	1	
Rat	IgG2a	mono	GFP	Antikoerper-online.de	ABIN398304	P1	1.8	1	
Humanized	IgG1	mono	HER2	gift of R. Schibli	Trastuzumab	Atto647N	2.2	1	Atto647N-cadaverine was used
Humanized	IgG1	mono	L1CAM	gift of R. Schibli	chCE7	AF488	1.9	1	
Humanized	IgG1	mono	L1CAM	gift of R. Schibli	chCE7	AF647	1.3±0.1	3	
Guinea pig	IgG	poly	n.a.	Jackson	006-000-002	AF647	2.0±0.1	2	

Rabbit	IgG	poly	Clathrin HC	Abcam	ab21679	AF647	0.2	1	unclear suitability
Rabbit	IgG	poly	Human IgG H+L	Jackson	309-005-003	AF647	0.2±0.2	3	unclear suitability
Goat	IgG	poly	Guinea pig IgG H+L	Jackson	106-005-003	AF647	1.1±0.1	2	unclear suitability

Table S2. Optimization of functionalization. Evaluation of the DOL of antibody-Alexa Fluor 647 conjugates using different molar excess of labeling reagents.

Antibody	Molar excess H_2N-PEG_3-N_3	Molar excess DIBO-AF647	DOL
Donkey-anti rabbit	80	20	1.9
Donkey-anti mouse	80	20	2.0
Donkey-anti mouse	80	10	2.1
Donkey-anti rabbit	300	10	1.7

Table S3. Intramolecular distances within mouse immunoglobulin IgG2a (PDB: 1IGT).

Part	Start		End		Length (nm)
	chain	*residue*	*chain*	*residue*	
CDR - Hinge	D	Tyr102	D	Thr221	6.819
CDR - Hinge	B	Tyr102	B	Thr221	7.157
CDR - Hinge	D	Ile51	D	Thr221	6.527
CDR - Hinge	B	Ile51	B	Thr221	6.669
Hinge – Glutamine	D	Thr221	D	Glu295	5.523
Hinge – Glutamine	B	Thr221	B	Glu295	3.217
Hinge – Glutamine	D	Thr221	B	Glu295	3.898
Hinge – Glutamine	B	Thr221	D	Glu295	6.072
Hinge – proximal Fc	B	Thr221	B	Pro231	2.520
Hinge – distal Fc	B	Thr221	B	Glu420	7.239

Table S4. Parameters of the immunolabeling model used for Monte-Carlo simulations. For illustrations, see Figure 3a,b and Supplementary Figure S5. Parameter choices are detailed in the Methods section.

Structure	Segment	Parameter	Value/Range
Microtubule	1° Epitope on α-tubulin	y_0 (nm)	$10.5 + 2.75 \cdot \cos(\phi_0)$
		z_0 (nm)	$2.75 \cdot \sin(\phi_0)$
		ϕ_0	$\in$ [-45°…45°]
1° Antibody	CDR-Hinge	L_1 (nm)	$\in$ [6.527, 6.669, 6.819, 7.157]
		ϕ_1	$\in [\phi_0-\delta\phi \ldots \phi_0+\delta\phi]$
		θ_1	$\in [-\delta\phi \ldots \delta\phi]$
		$\Delta\phi$	22.5°
	Hinge-Glu	L_2 (nm)	$\in$ [3.217, 3.898, 5.523, 6.072]
		ϕ_2	$\in$ [15°…127.6°]
		θ_2	$\in$ [0°…360°]
	Hinge-2° Epitope	L_2 (nm)	$\in$ [2.520…7.239]
2° Antibody	CDR-Hinge	L_3 (nm)	$\in$ [6.527, 6.669, 6.819, 7.157]
		ϕ_3	$\in [90°-\delta\phi \ldots 90°+\delta\phi]$
		θ_3	[0 … 360°]
	Hinge-Glu	L_4 (nm)	[3.217, 3.898, 5.523, 6.072] (nm)
		ϕ_4	[15°…127.6°]
		θ_4	[0°…360°]
Reporter	Linker	L_5 (nm)	$\in$ [0.1…0.63]
		ϕ_5	$\in$ [10°…170°]
		θ_5	$\in$ [-80°…80°]
Steric restriction	Non-neighboring segments	d_{min} (nm)	3.0

Table S5. Vendor and approximate pricing information

Material	Vendor	Product number	Purchase price	Price per labeling reaction (50 µg IgG)
Microbial transglutaminase	Zedira	T001	270 € for 25 U	6.50 €
PNGase	Roche	11365193001	324 € for 100 U	0.98 €
H_2N-PEG_3-N_3	Jena Bioscience	CLK-AZ101-100	122 € for 100 mg	0.01 €
DIBO-Alexa Fluor 647	ThermoFisher	C20022	516 € for 0.5 mg	3.57 €
DBCO-ssDNA	Biomers.net	n.a. (Scale L)	86 € for ca. 0.2 µmol	1.43 €
Centrifugal concentrator	GE Healthcare	Vivaspin 500	105 € for 25	8.40 €
		Total costs	*1'588 € once*	*19.46 € or 17.32 € (AF647 or P1)*

Table S6. DNA sequences used for DNA-PAINT.

Name	Sequence	source
P1 imager	5'- CTAGATGTAT-Atto655	MWG Eurofins Genomics
P1 handle	DBCO-TTATACATCTA-3'	Biomers.net

Supplementary References

(1) Li, Y.; Mund, M.; Hoess, P.; Deschamps, J.; Matti, U.; Nijmeijer, B.; Sabinina, V. J.; Ellenberg, J.; Schoen, I.; Ries, J. Real-Time 3D Single-Molecule Localization Using Experimental Point Spread Functions. *Nat. Methods* **2018**, *15* (5), 367–369. https://doi.org/10.1038/nmeth.4661.

(2) Deschamps, J.; Rowald, A.; Ries, J. Efficient Homogeneous Illumination and Optical Sectioning for Quantitative Single-Molecule Localization Microscopy. *Opt. Express* **2016**, *24* (24), 28080. https://doi.org/10.1364/OE.24.028080.

(3) Deschamps, J.; Ries, J. EMU: Reconfigurable Graphical User Interfaces for Micro-Manager. *BMC Bioinformatics* **2020**, *21* (1), 1–7. https://doi.org/10.1186/s12859-020-03727-8.

(4) Ries, J. SMAP: A Modular Super-Resolution Microscopy Analysis Platform for SMLM Data. *Nat. Methods* **2020**, *17* (9), 870–872. https://doi.org/10.1038/s41592-020-0938-1.

(5) Szenczi, Á.; Kardos, J.; Medgyesi, G. A.; Závodszky, P. The Effect of Solvent Environment on the Conformation and Stability of Human Polyclonal IgG in Solution. *Biologicals* **2006**, *34* (1 SPEC. ISS.), 5–14. https://doi.org/10.1016/j.biologicals.2005.06.007.
